# The radiosensitizing effect of Ku70/80 knockdown in MCF10A cells irradiated with X-rays and p(66)+Be(40) neutrons

**DOI:** 10.1186/1748-717X-5-30

**Published:** 2010-04-27

**Authors:** Veerle Vandersickel, Monica Mancini, Jacobus Slabbert, Emanuela Marras, Hubert Thierens, Gianpaolo Perletti, Anne Vral

**Affiliations:** 1 Department of Basic Medical Sciences, Ghent University, De Pintelaan 185, 9000 Gent, Belgium; 2 Department of Structural and Functional Biology, Laboratory of Toxicology and Pharmacology, Università degli Studi dell' Insubria, via A. Da Guissano 10, 21052 Busto Arsizio, Italy; 3 NRF iThemba LABS (Laboratory for Accelerated Based Sciences), PO box 722, 7129 Somerset West, South Africa; 4 Department of Medical Imaging and Clinical Oncology, University of Stellenbosch, South Africa

## Abstract

**Background:**

A better understanding of the underlying mechanisms of DNA repair after low- and high-LET radiations represents a research priority aimed at improving the outcome of clinical radiotherapy. To date however, our knowledge regarding the importance of DNA DSB repair proteins and mechanisms in the response of human cells to high-LET radiation, is far from being complete.

**Methods:**

We investigated the radiosensitizing effect after interfering with the DNA repair capacity in a human mammary epithelial cell line (MCF10A) by lentiviral-mediated RNA interference (RNAi) of the Ku70 protein, a key-element of the nonhomologous end-joining (NHEJ) pathway. Following irradiation of control and Ku-deficient cell lines with either 6 MV X-rays or p(66)+Be(40) neutrons, cellular radiosensitivity testing was performed using a crystal violet cell proliferation assay. Chromosomal radiosensitivity was evaluated using the micronucleus (MN) assay.

**Results:**

RNAi of Ku70 caused downregulation of both the Ku70 and the Ku80 proteins. This downregulation sensitized cells to both X-rays and neutrons. Comparable dose modifying factors (DMFs) for X-rays and neutrons of 1.62 and 1.52 respectively were obtained with the cell proliferation assay, which points to the similar involvement of the Ku heterodimer in the cellular response to both types of radiation beams. After using the MN assay to evaluate chromosomal radiosensitivity, the obtained DMFs for X-ray doses of 2 and 4 Gy were 2.95 and 2.66 respectively. After neutron irradiation, the DMFs for doses of 1 and 2 Gy were 3.36 and 2.82 respectively. The fact that DMFs are in the same range for X-rays and neutrons confirms a similar importance of the NHEJ pathway and the Ku heterodimer for repairing DNA damage induced by both X-rays and p(66)+Be(40) neutrons.

**Conclusions:**

Interfering with the NHEJ pathway enhanced the radiosensitivity of human MCF10A cells to low-LET X-rays and high-LET neutrons, pointing to the importance of the Ku heterodimer for repairing damage induced by both types of radiation. Further research using other high-LET radiation sources is however needed to unravel the involvement of DNA double strand break repair pathways and proteins in the cellular response of human cells to high-LET radiation.

## Background

It is generally accepted that the effectiveness of ionizing radiation depends on the quality of the radiation beam. Densely ionizing, high-linear energy transfer (LET) types of radiation are biologically more effective than sparsely ionizing, low-LET types of radiation at inducing cell lethality for a given absorbed dose. This increased efficiency of inactivating cells by high-LET beams compared to low-LET beams is usually described by the relative biological effectiveness (RBE).

Among the various types of DNA damage, DNA double strand breaks (DSBs) are considered the most cytotoxic lesions induced by ionizing radiation. As many types of high-LET beams, including neutrons, in general do not appear to induce more DSBs than low-LET radiation [1-7], it seems likely that the differences in biological effect are associated with the type of DSBs induced by radiations of differing LET and the mechanisms involved in the processing of those DSBs. It has been described that the degree of complexity of DNA DSBs and its possible association with other types of damage varies depending on the LET characteristics; therefore the biological repairability of DSBs may vary with radiation type [3,8,9].

In mammalian cells, the homologous recombination (HR) and nonhomologous end-joining (NHEJ) pathways are identified as the two main mechanisms involved in the repair of DSBs. The NHEJ pathway however is regarded as the major pathway for the repair of radiation-induced DSBs in mammalian cells [10,11]. One of the key-players in this pathway is the Ku heterodimer, a highly stable protein complex consisting of a 70 kDa and a 86 kDa polypeptide, better known as Ku70 and Ku80 [12,13]. The importance of the Ku70 and Ku80 proteins in DNA DSB repair after *low*-LET radiation is well demonstrated by the profound enhancement in radiosensitivity of both Ku80-defective mutant rodent cell lines (e.g. the xrs-5 and xrs-6 cell line) [14] and human cell lines expressing reduced levels of the Ku proteins [15-23]. To date however, the knowledge regarding the importance of the Ku heterodimer and the NHEJ repair mechanism in the cellular response to *high*-LET radiation, including high energy neutrons, is limited and diverging results were described when using cell survival as an endpoint to analyze radiosensitivity [3,5,24-28]. In these reports, cellular radiosensitivity was investigated in Ku-deficient rodent cell lines with a wide variety of high-LET radiation qualities (fast neutrons, α-particles, iron ions, carbon ions). When the high-LET beams used had mean LET values inferior to 100 keV/μm, the majority of these studies reported similar RBE values in the repair-deficient and -proficient cell lines [3,5,24] pointing to an involvement of the Ku protein in the repair of the radiation-induced damage. When the radiation quality of the high-LET beam was superior to 100 keV/μm, RBE values close to or equal to 1 in repair-deficient cell lines were observed [3,25-27], indicating no major involvement of the NHEJ mechanism in the repair of high-LET radiation-induced damage. However, contradictory observations [28] and the lack of studies conducted with *human *Ku-deficient cell lines suggests the importance of further research into the biological mechanisms involved in the cellular response to high-LET radiation, especially given the growing interest and use of high-LET radiation in radiotherapy [29,30].

In the present study, we investigated the role of the Ku heterodimer in the repair of DNA lesions induced by p(66)+Be(40) neutrons (mean LET ~20 keV/μm) and 6 MV X-rays. After knockdown of the Ku heterodimer by lentiviral-mediated RNA interference (RNAi) of Ku70 in a human mammary epithelial cell line (MCF10A), cellular radiosensitivity was measured using a crystal violet cell proliferation assay, while chromosomal radiosensitivity was evaluated using the micronucleus (MN) assay.

## Methods

### Cell Culture

MCF10A cells, spontaneously immortalized human breast epithelial cells, were cultured as monolayers in DMEM/F12-Ham supplemented with 5% horse serum, growth factors and antibiotics [23] in a humidified 5% CO_2 _incubator at 37°C. To generate a repair-deficient cell line, MCF10A cells were transduced with lentiviral particles harboring DNA sequences encoding for short hairpin RNA specific for Ku70 RNA interference (= Ku70i cells). As a control cell line, MCF10A cells were mock-transduced with 'empty' lentiviral particles (= LVTHM cells). More details can be found in Vandersickel et al. [23]. Protein expression silencing of Ku70 and Ku80 by western blot analysis was evaluated in Ku70i and LVTHM cells. When a stable knockdown was obtained, these cells were used for all *in vitro *radiation experiments.

### Radiation Experiments

#### Irradiation conditions

80% confluent cell cultures were trypsinized and plated at appropriate densities 2 h prior to irradiation. Duplicate cultures were irradiated at room temperature with either 6 MV X-rays or a clinical neutron beam which is produced by the reaction of 66 MeV protons on a Be target: p(66)+Be(40) [31]. The neutrons produced in this beam have a mean energy of 29 MeV and a mean LET of about 20 keV/μm. Within each experiment, one cell culture was also sham irradiated.

Neutron exposures were performed using a vertical beam directed downwards. Cultures were placed in a 29 × 29 cm^2 ^field on a 15 cm-thick backscatter block of perspex. Build-up material consisted of a 20 mm thick polyethylene layer. Under these conditions the γ-component in the beam is 6.9% and the total dose rate to the samples was ~0.4 Gy/min. The neutron beam was calibrated using a 0.5-cm^3 ^tissue equivalent ionization chamber. The neutron dose conformations at the irradiated position were done as part of the routine quality control measures used for daily radiation therapy.

X-ray irradiations were performed using a Philips SL 75-5 linear accelerator calibrated to use 6 MV X-rays. A vertical treatment field of 30 cm × 30 cm was used to irradiate cell samples in multiwell plates or cell culture flasks. A build-up plate of 20 mm polyethylene was used and cell samples were placed on a block of 15 cm thick Perspex.

### Crystal violet cell proliferation assay

As the colony forming ability of the LVTHM and Ku70i cells was inadequate to quantify radiation-induced damage, a cell proliferation method was used. Although the crystal violet cell proliferative assay yield parameters different from that obtained with the classic colony formation assay, the crystal violet staining method has been shown to reflect the relative radiosensitivities of different cell lines [32]. For this assay, 2500 cells were seeded in 24-well plates and exposed to doses ranging from 0 to 6 Gy of X-rays or 0 to 3 Gy p(66)+Be(40) neutrons. Cells were allowed to grow for several days until the control plates (0 Gy) nearly reached confluency. After fixation and staining with 0.01% crystal violet, optical density measurements of extracted dye served as a measure of cell growth. Cell survival at each dose point was expressed as a percentage of the control survival rate [23,32].

### Micronucleus assay

8 × 10^5 ^cells were seeded into T25 tissue culture flasks and exposed to doses ranging from 0 to 6 Gy of X-rays or 0 to 3 Gy of p(66)+Be(40) neutrons. Cytochalasin B (2.25 μg/ml) was added immediately after irradiations to block cytokinesis. Forty eight hours post-irradiation, cells were harvested by trypsinization. Cell fixation, staining and analysis of the samples were performed as previously described [33]. Micronuclei were scored by light microscopy in 1000 binucleated (BN) cells.

### Data analysis

Log cell surviving fractions (S) were fitted as a function of radiation dose (D) to a linear-quadratic equation as log_e_(S) = -αD - βD^2 ^(Graphpad Prism 4 software). Radiosensitivities were expressed in terms of the mean inactivation dose (MID). This parameter quantifies radiosensitivity in a one-dimensional parameter with units of dose (Gy). The mean inactivation dose is proportional to the area under the survival curve. The ratio MID_LVTHM_/MID_Ku70i _represents the corresponding dose modifying factor (DMF) and is used to evaluate the effect of the Ku70/80 knockdown on cell survival. To compare the effects of the radiation qualities (X-rays vs. neutrons), the RBE of the neutron beam is defined by the ratio of MID_X _to the MID_n_.

MN frequencies (Y) as a function of dose were best fitted for both radiation qualities to a linear-quadratic model Y = c+ αD+ βD^2^. The RBE generally used is given by the ratio of the X-ray dose to the neutron dose to obtain equal biological effects (iso-effect RBE). Because of the slightly different shapes of the two linear quadratic dose response curves, no single RBE value for fast neutrons with respect to X-rays, covering the whole dose range, can be given. Therefore isoeffect RBE values have been calculated for different doses by solving c_X_+ α_X_D_X_+ β_X_D^2^_X _= c_n _+ α_n_D_n_+ β_n_D^2^_n _for D_X _and substituting the result in the RBE expression [34]. This yields:

The DMF, to evaluate the effect of knocking down the repair proteins Ku70 and Ku80 on MN formation, can be calculated for different dose points in a similar way:

## Results

### Downregulation of the Ku heterodimer by RNAi of Ku70

**Figure 1:**
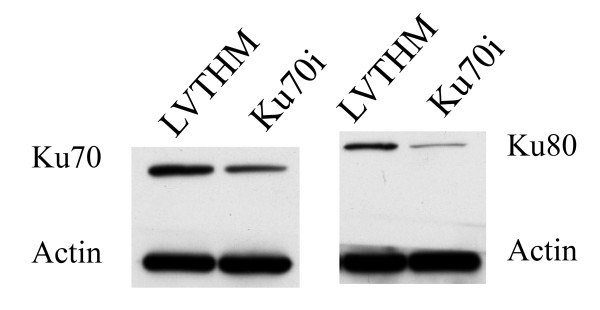
**Western blot of MCF10A cells after RNAi of Ku70**. Protein expression levels of the Ku70 and the Ku80 protein are shown in both the LVTHM (control cell line) and Ku70i (RNAi of Ku70) cell line. Actin was used as a protein loading control. RNAi of Ku70 caused downregulation of both the Ku70 and the Ku80 proteins.

Western blot analysis confirms (Figure 1) that RNAi of Ku70 causes a stable knockdown of the Ku70 protein. In addition, a stable knockdown of the Ku80 subunit is also observed. These findings are in agreement with several independent reports showing that loss or decrease of one of the subunits resulted in a significant decrease in the steady state level of the other (for a review, see [23]). It seems that each subunit is required to stabilize the other. This is not unexpected in view of their function as a heterodimer in the NHEJ repair pathway [12,13].

### Crystal violet cell proliferation assay

**Figure 2:**
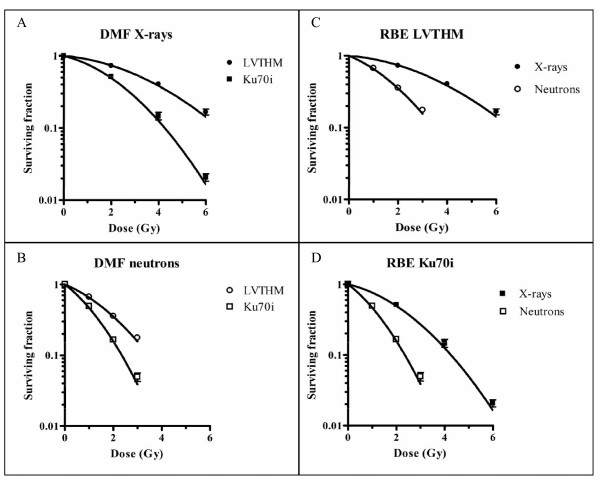
**Cell survival curves after exposure of Ku70i and LVTHM MCF10A cells to X-ray doses ranging from 0 to 6 Gy or neutron doses from 0 to 3 Gy**. Cell survival was measured using a crystal violet cell proliferation assay. Log surviving fractions were fitted as a function of dose using the linear quadratic equation. Each data point represents the mean ± SEM of 4 experiments. (A) and (B) show the effect of Ku70/80 knockdown on cell survival for X-rays and neutrons respectively. In (C) and (D) a comparison of the effect of the radiation qualities in cells with wild type levels (LVTHM cells) and lower expression levels of Ku70/80 (Ku70i cells) respectively, is presented.

Our results (Figure 2, Table 1) show a dose dependent decrease in cell survival, which is more pronounced in the repair deficient Ku70i cell line. Radiosensitization is observed for both X-rays (Figure 2A) and neutrons (Figure 2B). After X-ray irradiation, the mean inactivation dose decreased from 3.60 Gy for the mock-transduced LVTHM cells to 2.22 Gy for the Ku70i cells, resulting in a DMF of 1.62. After neutron irradiation, a decrease in the mean inactivation dose of 1.74 Gy for the LVTHM cells to 1.14 Gy for the Ku70i cells is observed. This represents a DMF of 1.52.

**Table 1:** Survival parameters and MID for LVTHM and Ku70i MCF10A cells following exposure to neutrons and X-rays.

Radiation type		*α*	*β*	MID
p(66)+Be(40) neutrons	LVTHM	0.33	0.097	1.74
	Ku70i	0.60	0.161	1.14
X-rays	LVTHM	0.07	0.043	3.60
	Ku70i	0.19	0.083	2.22

The RBE of the neutron beam observed with LVTHM cells, calculated using the ratio of the mean inactivation doses for X-rays and neutrons, is 2.07. The resulting RBE for the Ku70i cells is very similar (1.95).

### Micronucleus assay

**Figure 3:**
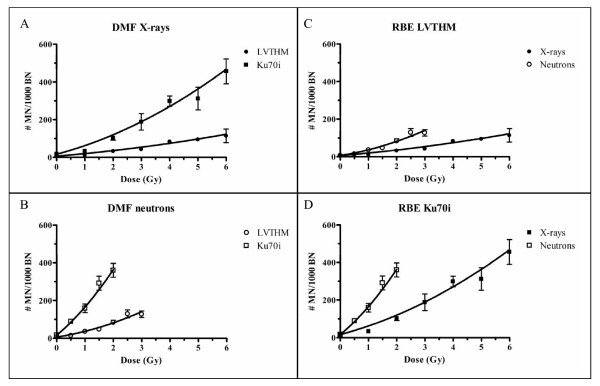
**Dose response curves of # MN/1000 BN cells after exposure of Ku70i and LVTHM MCF10A cells to X-ray doses ranging from 0 to 6 Gy or neutron doses from 0 to 3 Gy**. MN frequencies (Y) as a function of dose (D) were fitted to a linear-quadratic model Y = c+ αD+ βD^2^. Each data point represents the mean ± SEM of 2 experiments. The number of micronuclei at doses of 2 and 4 Gy X-rays and doses of 1 and 2 Gy neutrons represents the mean ± SEM of 9 experiments. (A) and (B) show the effect of Ku70/80 knockdown on MN formation for X-rays and neutrons respectively. In (C) and (D) a comparison of the effect of the radiation qualities in cells with wild type levels (LVTHM cells) and lower expression levels of Ku70/80 (Ku70i cells) respectively, is presented.

Dose response curves obtained after X-ray and neutron exposure show a dose dependent linear quadratic increase in micronuclei frequencies for both the Ku70i and LVTHM cell lines (Figure 3, Table 2). At each dose a higher MN yield was observed for the Ku70i cells compared to the mock-transduced LVTHM cells and this for both types of radiation. The DMFs, calculated for X-ray doses of 2 and 4 Gy are 2.95 and 2.66 respectively. After neutron irradiation, the DMFs for doses of 1 and 2 Gy are respectively 3.36 and 2.82.

**Table 2:** Fitted linear quadratic coefficients for neutrons and X-rays obtained after MN evaluation in LVTHM and Ku70i MCF10A cells.

Radiation type		*α*	*β*	c*
p(66)+Be(40) neutrons	LVTHM	23.54	7.08	6.33
	Ku70i	124.5	24.29	16.65
X-rays	LVTHM	12.36	1.17	6.33
	Ku70i	39.89	5.88	16.65

* c values represent spontaneous number of MN/1000 BN cells

Calculated RBE values for a neutron dose of 1 and 2 Gy are 2.07 and 2.16 for the LVTHM cells. For the Ku70i cells RBE values of 2.67 and 2.5 respectively are obtained.

## Discussion

Although an enhancement in radiosensitivity to low-LET radiation in Ku-deficient cells is well described, less is known about the effects of Ku-deficiency in the cellular response of *human *cells after exposure to high-LET radiation. In the present study, we investigated the role of the Ku heterodimer in the response of human breast epithelial MCF10A cells after exposure to 6 MV X-rays and p(66)+Be(40) neutrons. To this aim, cellular and chromosomal radiosensitivity were assessed in a control MCF10A cell line, and in a Ku70-knockdown derivative subline, obtained by RNA interference of Ku70.

The cell proliferation assay, used to assess cellular radiosensitivity, showed a RBE value of 2.07 in mock-transduced LVTHM cells. This finding, in agreement with other literature data [32,35], demonstrates that p(66)+Be(40) neutrons are indeed more effective per unit absorbed dose than X-rays in inactivating cell proliferation. A similar RBE value of 1.95 was found for repair deficient Ku70i cells, indicating a similar effectiveness of this neutron beam relative to X-rays with respect to inactivating cell proliferation in both repair-proficient and -deficient cell lines.

Marked differences observed in the cellular radiation response between the mock-transduced LVTHM and Ku70i cells further implicate that a partial knockdown of Ku results in an increase in radiosensitivity and this for both radiation qualities. DMFs of 1.62 and 1.52 were recorded following treatment with both 6 MV X-rays and p(66)+Be(40) neutrons, respectively. Interestingly, the observation that DMFs for both radiation treatment modalities were comparable demonstrates that the Ku heterodimer is as important for repairing radiation damage induced by 6 MV X-rays (mean LET < 1 keV/μm) and p(66)+Be(40) neutrons (mean LET ~20 keV/μm).

The MN assay was performed to assess chromosomal radiosensitivity in our cell model. Micronuclei are predominantly acentric chromosomal fragments resulting mainly from misrepaired DNA DSBs by the NHEJ pathway [36]. Results obtained with the MN assay in this study, showing DMFs that are in the same range for both neutrons and X-rays, confirm a similar importance of the NHEJ pathway and the Ku heterodimer for repairing DNA damage induced by both X-rays and high energy neutrons.

In summary, as the average LET of p(66)+Be(40) neutrons is about 20 keV/μm, these results are supporting the hypothesis of Britten et al. [3] who argued that several components of the DNA sensing/repair machinery may be of major relevance for the cellular response to low-LET as well as high-LET radiation when the latter have a mean LET value inferior to 100 keV/μm, while they would be of less importance for the repair of more complex lesions induced by radiation with LET values superior to 100 keV/μm. Because this hypothesis was based on data derived from experiments with Ku-defective rodent cell lines, our results give a first indication that the conclusions of Britten et al. may be extended to human cell lines. However, additional research using high-LET radiation beams with differing LET values is required to draw more general conclusions.

In addition, our findings are also interesting in the frame of the clinical use of both low- and high-LET radiation beams, such as clinical neutron [30] and carbon ion beams [29]. Despite recent remarkable progress in the efficacy of radiotherapy, cellular resistance to radiotherapy is still a significant component of tumor treatment failure. The ability to repair DNA damage is probably the most important determinant of resistance to ionizing radiation [37]. Therefore, reduction of the capacity of tumor cells to repair DSBs through targeted gene therapy mediated inactivation of DSB repair proteins may represent a promising strategy to enhance radioresponsiveness of neoplastic tissues and to increase radiation-induced tumor eradication rates [38,39].

## Conclusions

Our results show that partial knockdown of Ku, one of the key proteins involved in the NHEJ pathway for DNA DSB repair, enhances the radiosensitivity of human MCF10A cells to both 6 MV X-rays and p(66)+Be(40) neutrons. Dose modifying factors are very similar, irrespective of radiation quality, which demonstrates the importance of the Ku heterodimer for repairing radiation damage induced by both low-LET X-rays and high energy neutrons. Although additional research is required, these results provide evidence that selective modulation of the repair capacity of cells in tumor and normal tissues may represent a future strategy to enhance the effects of radiotherapy using X-rays or high energy neutrons. These results may also be equally applicable to carbon ion therapy, that is currently under development in both Europe and Japan [29].

## Competing interests

The authors declare that they have no competing interests.

## Authors' contributions

VV drafted the manuscript and performed all the radiation experiments together with MM. JS helped to outline and supervise the radiation experiments, which were all performed at iThemba LABS. EM and GP were responsible for the design, development and production of the lentiviral vectors and RNAi experiments. HT helped in the analysis and the discussion of the data. AV coordinated the study and contributed to the drafting of the manuscript.

All authors read and approved the final manuscript.
